# Fiber Reinforced Compressed Earth Blocks: Evaluating Flexural Strength Characteristics Using Short Flexural Beams

**DOI:** 10.3390/ma14226906

**Published:** 2021-11-16

**Authors:** Peter Donkor, Esther Obonyo, Christopher Ferraro

**Affiliations:** 1 M.E. Rinker School of Construction Management, University of Florida, Gainesville, FL 32611, USA; 2 School of Engineering Design, Pennsylvania State University, State College, University Park, PA 16802, USA; eao4@psu.edu; 3 Engineering School of Sustainable Infrastructure & Environment, University of Florida, Gainesville, FL 32611, USA; ferraro@ce.ufl.edu

**Keywords:** compressed earth blocks, polypropylene fibers, equivalent flexural strength, residual strength, flexural toughness

## Abstract

There are ongoing research efforts directed at addressing strength limitations of compressed earth blocks (CEB) that inhibit their deployment for structural applications, particularly in areas where masonry systems are regularly subjected to lateral loads from high winds. In this paper, the authors focus specifically on the extent to which polypropylene (PP) fibers can be used to enhance the flexural performance of CEB. Cementitious matrices used for CEB production exhibit low tensile and flexural strength (brittle) properties. This work investigates plain (unreinforced) and fiber-reinforced specimens (short flexural beams) with fiber mass content of 0.2, 0.4, 0.6, 0.8, and 1.0% and ordinary Portland cement (OPC) content of 8%. The influence of the inclusion of fiber was based on tests conducted using the Standard Test Method for Flexural Performance of Fiber-Reinforced Concrete (ASTM C1609). Material properties that were quantified included first-peak strength, peak strength, equivalent flexural strength, residual strength, and flexural toughness. There was an observed improvement in the performance of the soil-fiber matrixes based on these results of these tests. It was also observed that when the fiber content exceeded 0.6% and above, specimens exhibited a deflection- hardening behavior; an indication of improvement in ductility. An equivalent flexural strength predictive model is proposed.

## 1. Introduction

Earthen construction materials and techniques have existed for centuries. Approximately 30% of all the United Nations Educational, Scientific and Cultural Organization (UNESCO) World Heritage Sites utilize earth as a building material; a testament to the durability of earthen structures when properly constructed and maintained [1]. In spite of the global use of earth as a building material, there are real and perceived limitations to its structural use [2,3]. Some of the identified factors limiting the structural use of earthen masonry globally include relatively low strength properties compared to concrete-based blocks and burnt bricks, significant variations in performance depending on soil type used, manufacturing methods, and climatic conditions [2,4,5].

The modern use of earth in the form of compressed earth blocks (CEBs) can be traced back to 1950s [6]. CEBs are produced by mixing soil with a stabilizer, which is then moistened and compressed either manually or mechanically in a mold to produce blocks [6,7]. It has been observed that engineered CEBs can outperform adobe with respect to strength, durability, and embodied carbon [8]. Considered as an “appropriate technology” by the United Nations, CEB production is small scale, labor intensive, sustainable, and requires simple operating technology and maintenance. This makes CEBs a well-suited material for providing affordable housing units [8,9,10,11]. Despite the strength improvement achieved through using CEBs over other traditional forms of earthen construction, they are still more brittle and weaker in bending and compression in comparison to concrete masonry units (CMU) and fired bricks [4]. Their compressive strength values that range between 2.0 to 5.0 MPa, which is significantly lower than concrete masonry units and burnt bricks, which average between 13.10 MPa and 17.24 MPa, respectively [4,7,10].

Some of the disadvantages of CEBs and CEB technology is poor resistance to moisture when not properly constructed and the lack of standardized procedures for production and construction. Where codes and standards exist, they are very limited in scope [10,12]. Variability in soils from site to site means optimum mix proportions differ, thus complicating the issue of developing standards of practice and codes [13]. Obonyo et al. [2] identified specific properties of earthen blocks/masonry systems that need to be optimized to promote their use for structural applications. The properties identified include optimizing the strength of individual units, geometry of blocks, strength of mortar, deformation characteristics of blocks and mortar, joint thickness, suction of blocks, water retention of mortar, blockwork bonding, and workmanship.

The findings presented in this paper are part of a larger collaborative research effort aimed at developing engineered fiber-reinforced masonry systems for high wind regions. Some of the findings from the larger research effort have been presented in other publications [4,14,15,16,17]. Obonyo et al. [14] and Donkor et al. [15] reported a diminishing return in the strength of PP fiber reinforced CEBs when certain fiber thresholds are exceeded. The authors also reported that the length of the PP fibers used had an influence on the strength of the CEBs produced. Donkor and Obonyo [4] reported that PP fiber quantity influences CEB compressive strength, 3-point bending strength, and strain capacity. The authors further proposed a regression model for predicting PP fiber reinforced CEB compressive strength. Matrices used for specimen production in the study were hand mixed. Donkor and Obonyo [17] focused on the microstructural analysis of failed fiber reinforced CEBs and proposed best practices for PP fiber addition into CEB matrices. The flexural properties of the specimens were also analyzed. The research approach adopted is to improve strength properties at the block level before scaling up to the system level. This current paper analyzes the flexural properties of PP fiber reinforced matrices used for CEB production and focuses on developing an equivalent flexural strength predictive model. In non-conventional walling materials such as CEBs, flexural strength is an important design parameter with respect to lateral loads due to wind, floods, or other load scenarios that can cause out-of-plane bending [18]. Modulus of rupture, also referred to as flexural strength, is one of the material properties used to assess the durability of earthen building materials [19]. For example, the State of New Mexico’s Earthen Building Materials Code requires a minimum modulus of rupture of 50 psi for earthen building materials. Earthen building materials as defined under the code include adobe, burned adobe, compressed earth blocks, rammed earth, or terrón [20].

Research into CEBs have been focused on alternate or supplementary stabilizers [21,22,23,24,25,26]; influence of soil types on performance [2,27,28,29]; hygroscopicity [23,30,31]; system level performance [18,32,33,34,35,36,37,38]; and the use of fibers as reinforcement.

Widely seen as an attractive building material particularly in the global south, the more modern forms of earthen masonry systems are attracting attention from sustainable building movements within developed countries in an attempt at finding more ecologically friendly building materials [35,39]. For example, the Mortenson Center in Engineering for Developing Communities at the University of Colorado, USA, collaborated with the Apsaalooke Nation Housing Authority to put up affordable houses using CEBs for the Crow Tribe in the state of Montana, USA [40,41].

### 1.1. Outstanding Issues in the Use of Fiber in Earthen Masonry

Earth, which has been stabilized with a binding agent, such as Portland cement, is an acceptable building material with adequate strength in compression. Unfortunately, it is brittle with low tensile strength, which often leads to poor resistance to bending and the development of tensile cracks in response to external forces [42,43] There is historic precedence in ancient civilization of enhancing the tensile strength of unfired earthen masonry units and plaster using naturally occurring fibers such as straws and horsehair [44]. Such practices have been adopted in modern earthen masonry units. This can be accomplished using naturally occurring plant and animal fibers as well as synthetic fibers to reinforce soils for CEB production [6]. As observed by [43], such use of fibers can improve ductility, which in the wake of a disaster will delay catastrophic failure, thereby giving building occupants time to escape, as opposed to facing the risk of injury of even fatality because of sudden failure [45].

Fibers that have been used for CEB production includes sisal fiber [21,46]; coconut fiber [2,23,47,48]), straw [49], polyethylene [29,43], and jute [50,51]. The findings of these studies indicated that desirable structural properties could be realized through incorporating either natural or synthetic fibers in earthen masonry units. It was also observed that untreated natural fibers could degrade when exposed to the highly alkaline environment that is created following the hydration of cement [44]. This has a direct bearing on the durability of these fibers. Some addition research is necessary to identify the optimal strategies for using these fibers [23]. When the synthetic fibers in question are derivatives of post–consumer plastic waste products, their performance as secondary reinforcement is subject to variations—this is a known problem associated with the re-use of waste material [52]. Such material variability coupled with the inherent variation in soil properties begets difficulty with respect to the isolation of CEB performance properties that are directly linked to specific fiber properties/attributes. For this reason, this research used commercially available PP fibers used for concrete production. The authors draw from the use of PP in concrete and geotechnical engineering applications. Historically, CEB advancement has benefited from techniques used in both industries [6,53,54].

### 1.2. Contributions from This Work

This research evaluated flexural properties such as first-peak strength, peak strength, equivalent flexural strength, residual strength, and toughness of soil-cement matrices used for CEB production. Cementitious matrices are quasi-brittle and have low tensile strength and strain capacities. A practical means of enhancing the performance of such matrices is the inclusion of various types of fibers to enhance ductility, strength, toughness, and resistance to impact loads [55]. In addition, the strength of fiber-reinforced CEBs is influenced by fiber type and quantity, soil type, stabilizer type and quantity, and level of compaction of the matrix, curing conditions, and testing procedures. Soil type, stabilizer type and quantity, and level of compaction also affect the strength properties and durability of CEBs. Mechanical properties such as compressive strength, flexural strength, and shear strength can therefore be enhanced if an optimal fiber-reinforcing ratio is identified and used [42,56]. The paper evaluated equivalent flexural strength (*f_e_*) at different fiber dosages and proposed the model to establish the correlation between equivalent flexural strength (*f_e_*), first–peak (*f*_1_), and fiber mass (*W_f_*). Equivalent flexural strength (*f_e_*) shows the effectiveness of fibers to bridge cracks and enhance the energy absorption ability of the reinforced matrices under loading. The first–peak (*f*_1_) strength depicts the flexural behavior of beams (both reinforced and unreinforced) up to the onset of cracks. The model is therefore to help predict how different fiber dosages influence how effective the fibers are in bridging cracks after the onset of cracking.

In general, synthetic fibers such as PP are known to improve concrete freeze–thaw resistance, impact resistance, and shrinkage cracking [57]. In cement-stabilized soils used for geotechnical applications, the inclusion of randomly distributed PP fibers increases unconfined compressive strength, residual strength, absorbed energy, ductility, splitting tensile strength, and flexural toughness of soil mixtures [58,59,60,61,62]. This suggests that there is a potential for using PP fibers in CEB production. Due to production differences between soil-cement matrices used in geotechnical applications and those used in CEB production, there is the need for an evaluation of the effect of PP fibers on the flexural performance of matrices for CEB production.

Recent findings from studies carried out on the inclusion of fibers into CEB matrices include: fiber surface conditions influence CEB strength [43] fiber length, aspect ratio, and quantity influence the strength and failure mode of CEBs [4,15,63] and there is a diminishing return in strength when certain fiber thresholds are exceeded [15,30,63]. Mechanical properties such compressive strength, flexural strength, and shear strength can therefore be enhanced if an optimal fiber-reinforcing ratio is identified and used [3,42,49,63]. The existing body of knowledge on CEB flexural performance is scarce compared to that of conventional walling materials. The work discussed in this paper is directed at contributing to this body of knowledge. Specimens for flexural performance evaluation were produced with 8% OPC and fiber mass content of 0.2, 0.4, 0.6, 0.8, and 1.0%. Economic feasibility was outside the scope of this study.

Based on UNEP (ND), sixty million plastic drinking bottles are purchased every hour. UNEP also estimates that across the world, we deploy 5 trillion single-use plastic bags. Fifty percent of the existing plastics are usually discarded after a single use. Developing viable pathways for re-using this plastic to enhance the structural properties of CEBs could address the existing environmental sustainability problem of plastic pollution.

## 2. Experimental Program

### 2.1. Material Properties

**Figure 1:**
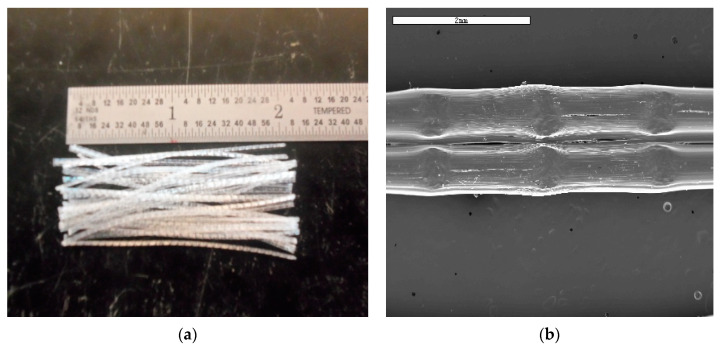
PP fibers: (**a**) photograph of fibers, (**b**) SEM image of fiber showing cross-linked filaments.

The materials used in the production of specimens included local soil (from the North-Central Florida region), OPC, and commercially available macro synthetic PP fibers typically used for OPC production. The fibers were 54 mm long with an equivalent diameter of 0.82 mm, an aspect ratio of 67, tensile strength of 585 MPa, and specific gravity of 0.91. Individual fiber strands (Figure 1) are composed of two circular filaments that are cross-linked into a single “stick–like” fiber. They have an embossed surface with depths from peak to valley of approximately 0.005 to 0.006 mm. The cross-linking and embossment provide mechanical anchorage between the fiber and matrices. The fibers have been successfully used in other applications to improve flexural toughness, impact resistance, residual strength, and durability [64].

The physical properties of the soil used in this study are presented in Table 1. The grain size distribution of the soil was determined using the American Association of State Highway and Transportation Officials (AASHTO) soil classification system M 145/ASTM D3282.

### 2.2. Test Specimen Preparation and Testing

Dry soil was passed through a manual sifter with a 3.40 square mm mesh to remove large particles prior to the matrix mixing of the soil and OPC. Mixing was done using a Kushlan 6 cubic feet capacity concrete mixer (Kushlan Products, Houston, TX, USA). The PP fibers were gradually introduced into the dry mix in batches at a rate of 0.045 kg every one minute. The fiber–reinforced matrices were produced with fibers at 0.2, 0.4, 0.6, 0.8, and 1.0 mass fractions. OPC content and water-cement-ratio was kept at 8.0% and 0.17, respectively. After the last batch of fibers was introduced, mixing continued for an additional 3 min. At this point, the dry mix was observed to be uniform and consistent, with the fibers well dispersed. Water was then added gradually and after 10 min, the wet mixture was visually deemed to be uniform and thoroughly mixed with the fibers well dispersed.

**Figure 2:**
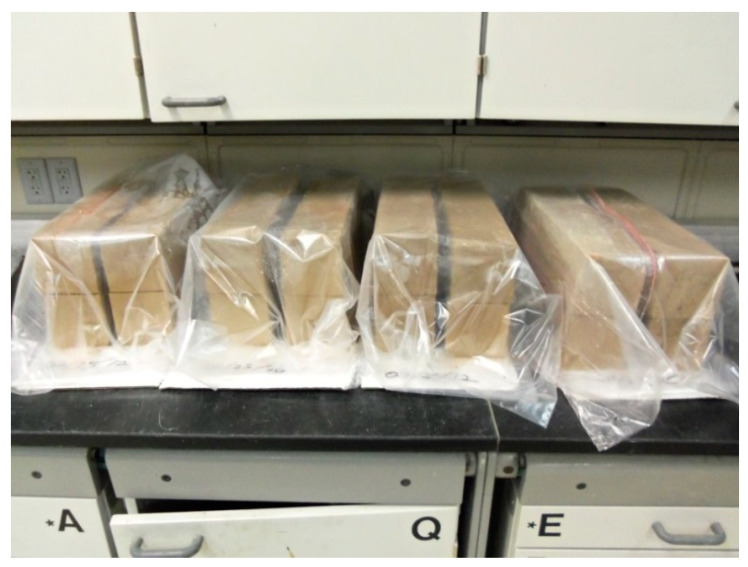
Initial cure of CEBs under plastic sheets.

A heavy-duty steel mold lined with form board was filled with matrix, covered with a form board lid, and compressed using a Test Mark CM-500 series compression machine (Test Mark Industries, East Palestine, OH, USA) with a maximum compression capacity of 2224 kN. Specimens used in this study were short flexural beams produced with soil cement and, where applicable, PP fibers. The specimens were produced using 8.62 kg of matrix in an effort to minimize variations in specimen densities. Each specimen was subjected to compaction at a rate of 223 N/min up to a maximum force of 1.6 MPa. The nominal dimensions of specimens produced were 413 mm (length) × 102 mm (width) × 102 mm (height). Five specimens were produced for each mix design. The specimens were moist-cured (sprayed with water) and stored under plastic sheets for the first 7 days (Figure 2) and kept under the plastic sheets for the next 21 days without further curing [4,6,34,35,36]. Testing was performed 28 days after production.

**Figure 3:**
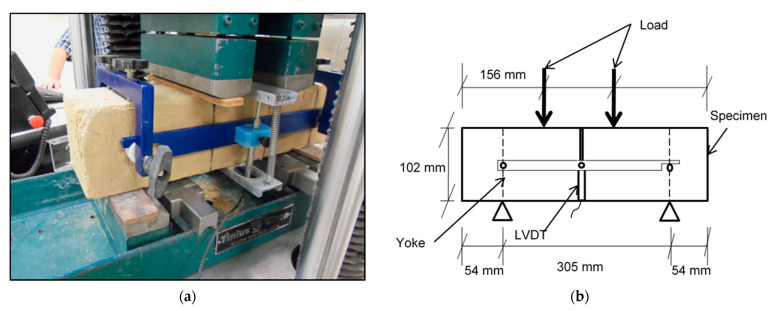
Test setup: (**a**) photograph, (**b**) schematic drawing.

There is no consensus on testing procedures for CEBs. CEB testing usually adopts methods, instrumentations, and procedures used for CMU and fired clay. For example, specimen geometry and aspect ratio are factors that influence test results of CEBs but are yet to be standardized. Neither has the extent of influence of such factors been agreed on [7]. To promote replicability, specimen sizing and flexural testing was performed according to the Standard Test Method for Flexural Performance of Fiber-Reinforced Concrete [65]. Testing was done using a Tinius Olsen compression machine with a maximum load capacity of 400 kN. The test setup consisted of a simply supported specimen with third-point loading, and a mounting jig (yoke) to hold linear variable displacement transducers (LVDTs) to record mid-span deflection. LVDTs with a stroke of ±17.8 mm were mounted on each side of the centerline of specimens to record mid-span deflection (Figure 3). Specimens were rotated through 90° from their casting position before testing to minimize the influence of casting direction on results. Leather shims were placed on the contact surface of specimens to provide an even surface and eliminate gaps during load application. Loading was deflection controlled at a rate of 0.25 mm/min.

**Figure 4:**
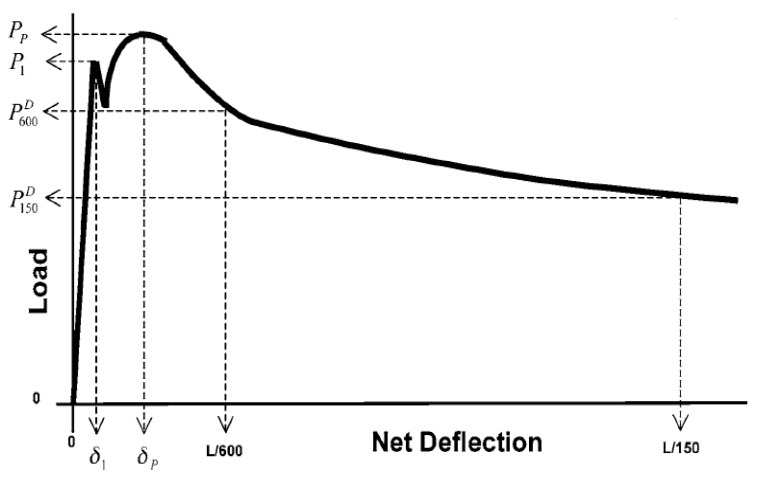
Schematic diagram of the load-deflection curve for calculating first-peak strength, residual strength, and toughness [65].

The load–deflection curves obtained during testing were used to calculate first-peak strength (*f*_1_) and peak strength (*f_p_*) (Equation (1)), equivalent flexural strength (*f*_e_) (Equation (2)), residual strength at deflections of *L*/600 ($f_{600}^{D}$) and *L*/150 ($f_{150}^{D}$) (Figure 4), and flexural toughness ($T_{b}$); area under load the load-deflection from 0 to *L*/150 (2.03 mm).

(1) $$f_{1/P}=\frac{P_{\left(1/P\right)}\;L}{bd²}$$

(2) $$f_{e}=\frac{T_{b}}{\delta_{tb}}\times \frac{L}{bd^{2}}$$

where; *f*_1_ = first–peak strength, *f_P_* = peak strength, P_1_ = first–peak load, *P_p_* = peak load, $P_{600}^{D}$ = residual load at net deflection of *L*/600, $P_{150}^{D}$ = residual load at net deflection of *L*/150, *b* = average width of specimen at the fracture, *d* = average depth of specimen at the fracture, $T_{b}$ = flexural toughness (Nmm), $\delta_{1}$ = net deflection at first-peak load, $\delta_{p}$ = net deflection at peak load, and $\delta_{tb}$ = net deflection of *L*/150.

## 3. Results and Discussion

### 3.1. Load–Deflection Response

**Figure 5:**
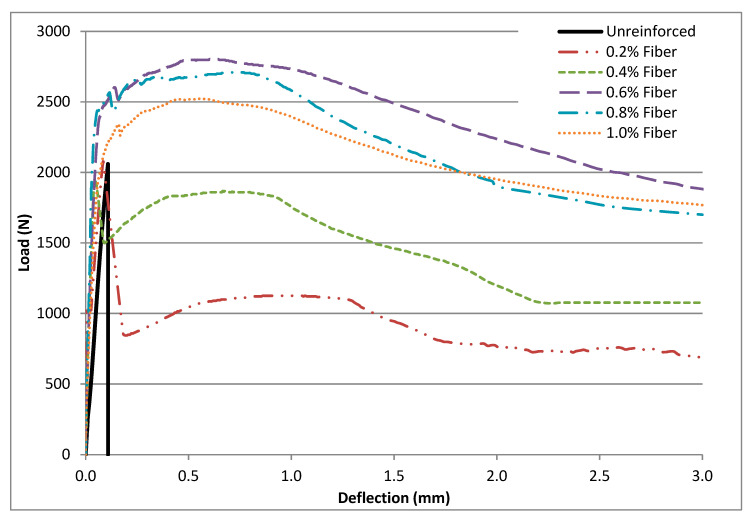
Typical load-deflection curves of specimens.

The load–deflection curves obtained during specimen testing are presented in Figure 5. With the unreinforced matrices, load increased linearly with increasing deflection until the onset of crack, which was the peak, with the load resulting in the catastrophic failure of the specimens. The catastrophic failure observed at peak load was an indication of the brittleness of the unreinforced matrices. The load–deflection response for the fiber-reinforced matrices was similar to the unreinforced matrices until first–peak load was reached. Specimens with 0.2% and 0.4% fiber content by mass experienced a sharp drop in load carrying capacity after first–peak load, which in this case occurred at first crack. Subsequent to continued loading, a gain in load carrying capacity was observed, which gradually dropped and flattened out. Observations made for the matrices with 0.4% fiber content were similar to that for 0.2% except that the drop in load carrying capacity after first–peak load was not as sharp for the matrices with 0.4% fiber content. Additionally, the regain in load carrying capacity of the 0.2% and 0.4% fiber reinforced specimens subsequent to first–peak load did not establish higher load capacity than what was recorded at first crack point. The load-deflection behavior observed is known as deflection-softening. As the fiber content within the specimens increased, the drop in load bearing capacity subsequent to the first crack point decreased; the matrices started exhibiting a deflection (strain) hardening behavior (Figure 5). Matrices characterized by the deflection-hardening behavior exhibit a higher load carrying capacity after first-peak loading [66]. In concrete, strain hardening occurs at high volume (greater than 2%) fiber fractions [67]. In this research, the hardening behavior began at 0.6% fiber mass content (2.6% volume content). Load carrying capacity typically increased above what was recorded at first crack point at 0.6%, 0.8%, and 1.0% fiber content (Figure 5).

### 3.2. Flexural Performance

Flexural performance was determined by the first-peak strength (*f*_1_), peak strength (*f_P_*), equivalent flexural strength (*f_e_*), residual strength at deflections of *L*/600 ($f_{600}^{D}$) and *L*/150 ($f_{150}^{D}$), and flexural toughness ($T_{b}$) at a deflection *L*/150 (Table 2, Table 3, Table 4, Table 5, Table 6, Table 7 and Table 8). The first-peak (*f*_1_) strength depicts the flexural behavior of the specimens (both reinforced and unreinforced) up to the onset of cracks. Peak strength (*f_p_*) was calculated using the maximum load obtained from the load–deflection curves (Figure 4). For the unreinforced matrices and matrices with 0.2% and 0.4% fibers, first-peak strength was the same as peak strength. In contrast, matrices that exhibited deflection hardening (0.6%, 0.8%, and 1.0% fibers) had their peak strengths greater than their corresponding first-peak strengths by about 8%, 28%, and 11%, respectively. The results show that as fiber content increased, average peak strength increased up to a fiber content of 0.6%. After this, average first-peak strength and peak strength steadily decreased as fiber dosage was increased. This suggests that the increases in strength observed is more of a function of fiber content optimization and not directly related to fiber content increase [57].

Equivalent flexural strength ($f_{e}$) shows the effectiveness of fibers to bridge cracks and enhance the energy absorption ability of the reinforced matrices under loading. The $f_{e}$ accounted for the flexural strength up to a deflection of *L*/150 (2.03 mm). The interfacial and frictional bonds activated when fibers are pulled out of matrices (slipping and stretching) improves the bonding between fibers and matrices, resulting in a high post first-peak strength even at high deformation levels as was observed with the fiber-reinforced matrices [60,68]. The best performing mix in terms of $f_{e}$ was mix PP-0.6, which had an average $f_{e}$ 21% higher than the next best performing mix PP-0.8. Mix PP-0.2 had the worst performance in terms of average $f_{e}$, which was 60% lower than mix PP-0.6.

Flexural toughness ($T_{b}$) is a measure of the energy absorption capacity of a material—an important parameter in characterizing a material’s resistance to fracture when subjected to dynamic or impact loads. It is an important property of fiber–reinforced composites as it shows the ability to absorb energy after cracking [44]. As fiber content was increased from 0.2% to 0.4% and 0.6%, flexural toughness increased by 39% and 80%, respectively. When fiber content was increased from 0.6% to 0.8% and 1.0%, flexural toughness dropped by 17% and 7%, respectively. Mixes that showed deflection–hardening performed better in flexural toughness because they could absorb more energy [69].

Residual strengths $f_{600}^{D}$ at *L*/600 (0.50 mm) and $\;f_{150}^{D}$ at *L*/150 (2.03 mm) in Table 6 and Table 7 represent the ability of the fiber–reinforced specimens to sustain load after first crack at the specified deflections [68]. The fiber–reinforced specimens maintained some load carrying capacity after first crack. At fiber content of 0.2% and 0.4% (mixes PP-0.2 and PP-0.4), average residual strengths at a deflection of *L*/600 ($f_{600}^{D}$) were lower than their corresponding first–peak strength. At fiber content of 0.6%, 0.8%, and 1.0% (mixes PP-0.6, PP-0.8, and PP-1.0), the average residual strength at a deflection of *L*/600 ($f_{600}^{D}$) was higher than the corresponding first-peak strength. Mix PP-0.6 had the highest average residual strength ($f_{600}^{D}$), which was about 6% higher than its corresponding first–peak strength. The average residual strength of the fiber-reinforced specimens at a deflection of *L*/150 ($\;f_{150}^{D}$) was lower than their corresponding first-peak strengths. Mix PP-0.6 had the highest average residual strength $f_{150}^{D}$, which was about 22% lower than its corresponding first-peak strength.

### 3.3. Influence of Fiber Content on Strength

The development of strength properties of soil-cement-fiber mixes for earthen blocks largely depends on the formation of fiber-matrix, matrix-matrix, and fiber-fiber bonds [47]. These identified bonds are influenced by the strength of the unreinforced matrix and fiber type (tensile strength), dimension, surface condition, and quantity present. The number of contact points between fibers and matrices are responsible for transmitting stress—a large quantity of fibers therefore reduces strength of earthen blocks [42]. Increasing fiber content in CEB matrices can also result in an increase in the development of micro-fractures at soil–fiber interfaces of CEBs, leading to a reduction in strength [21]. This explains the declining trend in first–peak strength (*f*_1_), peak strength, equivalent flexural strength (*f*_e_), residual strength ($f_{600}^{D}$ and $f_{150}^{D}$), and flexural toughness ($T_{b}$) in mixes with more than 0.6% fiber content. As the percentage of fibers present in matrices was increased, there was more fiber–fiber interaction, which did not result in the formation of bonds and less of the bond forming fiber–matrix interaction. Due to the stiffness of the fibers used (stick-like nature), a rebound of fibers or extension from the matrix was observed after de-molding where fibers near the surface of specimens “stick out”. This rebound of fibers shifts soil particles, resulting in a reduction or lowering of the bond between fiber and matrices [70]. Increasing the percentage of fiber content increased the occurrence of this rebound effect of the fibers because as fiber content increased, the quantity of fibers per each block increased. Other researchers have reported similar decreasing trends in strength of earthen blocks after exceeding certain fiber content thresholds. These include 2.0% salvaged steel fibers [71], 0.75% sisal fiber [21], and 1.5% barley straw [49]. The coefficient of variation reported in this study was between 6.80% and 25.58%. Other studies have reported coefficients of variation of between 9.2% and 48.5% [72], between 5.2% and 36.5% [73], and between 16.52% and 29.26% [4].

**Figure 6:**
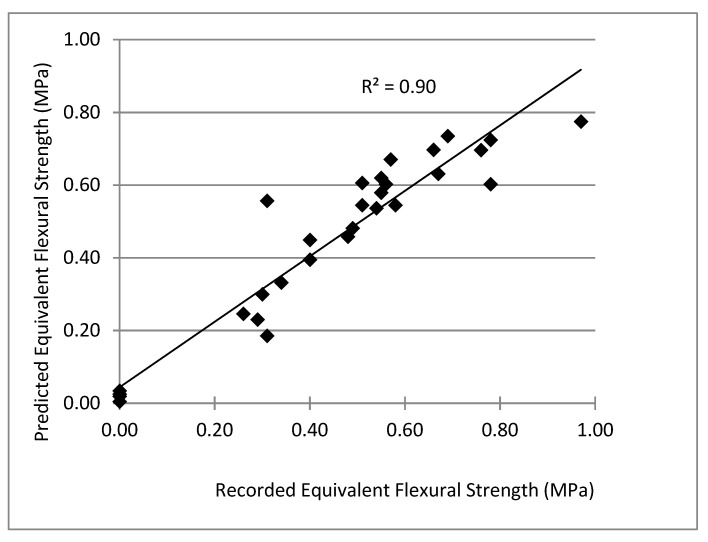
Predicted vs. experimentally recorded equivalent flexural strength (n = 30).

The lack of adequate and reliable empirical data that can be used to predict the structural performance of earthen construction materials limits the use of CEBs as a structural material [6,10,12]. For this research, statistical regression analysis was performed on the test results in an effort to create a model to predict equivalent flexural strength (*f_e_*) based on the first–peak strength and fiber mass fraction (Equation (3)). Table 9 summarizes the details of the model where the coefficients of the model were statistically significant at the 10% significance level as demonstrated by the *p*-values in Table 9. Figure 6 shows the relationship between the equivalent flexural strength (*f_e_*) predicted using Equation (3) and those calculated from the experimental results.

(3) $$f_{e}=-0.27+0.59f_{1}+1.07W_{f}-0.573W_{f^{3}}$$

where *f*_1_ = first—peak strength (MPa), and *W_f_* = the fiber mass (%).

### 3.4. Crack Patterns

**Figure 7:**
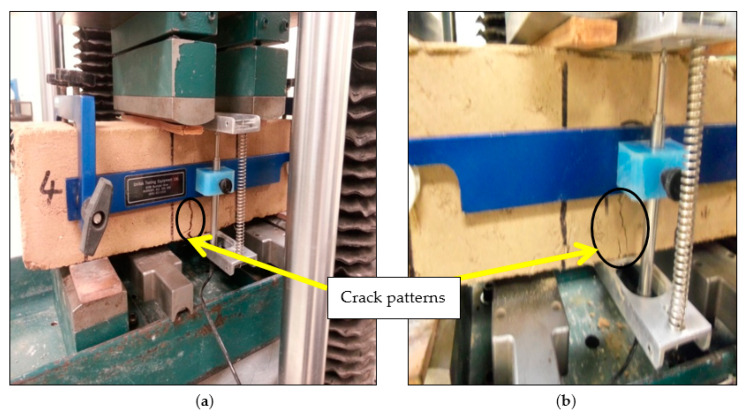
Typical crack patterns: (**a**) beams reinforced with 0.2 and 0.4% fibers, (**b**) beams reinforced with 0.6, 0.8, and 1.0% fibers.

The crack patterns that formed under flexural loading were observed during specimen testing. The plain matrix specimens underwent sudden failure at the onset of crack. Cracks in specimens reinforced with 0.2 and 0.4 fiber content were generally straight and coalesced from top to bottom in the middle third of specimens. Failure was mostly localized at a single crack (Figure 7a). The fibers resisted the formation of large crack widths. As fiber content increased to 0.6%, 0.8%, and 1.0%, failure was typically characterized by multiple cracks (Figure 7b). This multiple cracking phenomenon observed is the result of the stress–redistribution action of the fibers. After the formation of the first macro-crack, the load carried by the matrix was transferred to the bridging fibers. The fibers acting as a bridge, transfers the load back to the matrix through the fiber-matrix interface. This process leads to the redistribution of load into the brittle matrix, resulting in the development of another crack. As the process continues, multiple cracks develop and the load carrying capacity (strength) of composites can exceed the first cracking strength of the composite. The increase in load carrying capacity subsequent to the first crack, and associated multiple cracking for matrices with 0.6%, 0.8%, and 1.0% fiber content that was observed in this research is consistent with other research [69,74].

The deflection–hardening behavior associated with multiple cracking results in an increase in toughness, which is important for the serviceability and durability of structures under different loading conditions [69]. The toughness recorded for mixes that exhibited multiple cracking (PP-0.6, PP-0.8, and PP-1.0) was higher than mixes (PP-0.0, PP-0.2, and PP-0.4) that failed with a single crack. According to [74], a critical minimum fiber quantity is needed for multiple cracking to occur. In this research, multiple cracking started occurring at 0.6% fiber content. The observed failure patterns suggest that the inclusion of the fiber improved the ductility of the specimens.

## 4. Conclusions

This research was directed at assessing the extent to which PP fibers could be used to enhance the performance of earthen masonry units. The authors focused specifically on investigating the influence of the incorporation of fibers and fiber content on the flexural behavior of CEB matrices. Unreinforced and PP fiber–fiber reinforced specimens (short flexural beams) were produced with 8% OPC and 0.2, 0.4, 0.6, 0.8, and 1.0% fiber dosages by mass. The specimens were tested according to Standard Test Method for Flexural Performance of Fiber-Reinforced Concrete per ASTM C 1609. The strength of fiber-reinforced CEBs is influenced by fiber type and quantity, soil type, stabilizer type and quantity, and level of compaction of the matrix, curing conditions, and testing procedures. Within the limits of the experimental program used, the main conclusions have been outlined below:

- The inclusion of the PP fibers into the soil–cement matrices enhanced flexural performance as seen by the load-deflection response after initial crack, residual strength, flexural toughness, and equivalent flexural strength.
- At fiber content of 0.2 and 0.4%, there was a sharp drop in load carrying capacity after initial crack. A rebound in load carrying capacity was observed after the fibers were engaged. This was followed by a deflection-softening behavior.
- At fiber content of 0.6% and above, a deflection-hardening behavior was observed after a slight drop in load carrying capacity after the onset of crack.
- Fiber inclusion resulted in a high degree of load retention after first crack. The unreinforced matrices were brittle and experienced catastrophic failure.
- The fibers prevented catastrophic failure of the reinforced specimens even at large crack widths. The fiber-reinforced specimens deformed significantly after ultimate load without collapse.
- The failure patterns and response of the fiber–reinforced specimens during testing suggests an improvement in ductility as a result of PP fiber reinforcement.
- An equivalent flexural strength predictive model with first-peak strength and fiber mass content as variables was proposed.
- The optimal fiber mass content was determined to 0.6% based on test results. In subsequent research, the social, financial, and economic aspects of implementing the proposed use of PP as secondary reinforcement for engineered earthen masonry units should be investigated further.

## Figures and Tables

**Table 1:** Physical properties of soil.

Property	Composition
Liquid Limit (%)	33%
Plastic Limit (%)	(non-plastic)
Plasticity Index (%)	-
Sand (%)	87.3%
Clay (%)	12.2%
Silt (%)	1.5%
Optimum Moisture Content	9%
Maximum Dry Density	1784.5 kg/m^3^

**Table 2:** First peak strength (MPa).

Fiber Content	Specimens					Average	S.D. *	COV **
	1	2	3	4	5			
No Fiber	0.43	0.48	0.49	0.46	0.51	0.47	0.03	6.80
0.20%	0.49	0.41	0.66	0.51	0.60	0.54	0.10	18.19
0.40%	0.55	0.46	0.61	0.57	0.73	0.58	0.10	17.03
0.60%	0.80	0.76	0.71	0.89	0.76	0.78	0.07	8.54
0.80%	0.52	0.52	0.55	0.75	0.53	0.58	0.10	16.80
1.00%	0.60	0.54	0.54	0.68	0.52	0.58	0.07	11.52

* Standard deviation, ** Coefficient of variation.

**Table 3:** Peak strength (MPa).

Fiber Content	Specimens					Average	S.D. *	COV **
	1	2	3	4	5			
No Fiber	0.43	0.48	0.49	0.46	0.51	0.47	0.03	6.80
0.20%	0.49	0.41	0.66	0.51	0.60	0.54	0.10	18.19
0.40%	0.55	0.46	0.61	0.57	0.73	0.58	0.10	17.03
0.60%	0.87	0.76	0.75	1.04	0.81	0.84	0.12	14.12
0.80%	0.67	0.83	0.62	0.79	0.79	0.74	0.09	12.14
1.00%	0.63	0.60	0.62	0.73	0.55	0.63	0.07	10.57

* Standard deviation, ** Coefficient of variation.

**Table 4:** Equivalent flexural strength (MPa).

Fiber Content	Specimens					Average	S.D. *	COV **
	1	2	3	4	5			
0.20%	0.29	0.31	0.34	0.26	0.30	0.30	0.03	9.78
0.40%	0.40	0.40	0.49	0.48	0.31	0.42	0.07	17.16
0.60%	0.78	0.66	0.57	0.97	0.76	0.75	0.15	20.42
0.80%	0.56	0.78	0.55	0.69	0.51	0.62	0.11	17.91
1.00%	0.55	0.51	0.58	0.67	0.54	0.57	0.06	10.78

* Standard deviation, ** Coefficient of variation.

**Table 5:** Flexural toughness (Nmm).

Fiber Content	Specimens					Average	S.D. *	COV **
	1	2	3	4	5			
0.20%	2001	2156	2369	1813	2105	2088.80	204.37	9.78
0.40%	2765	2794	3425	3330	2192	2901.20	497.81	17.16
0.60%	5415	4588	3964	6804	5281	5210.40	1063.87	20.42
0.80%	3899	5423	3842	4804	3593	4312.20	772.14	17.91
1.00%	3843	3593	4082	4694	3750	3992.40	430.37	10.78

* Standard deviation, ** Coefficient of variation.

**Table 6:** Residual strength at *L*/150 (MPa).

Fiber Content	Specimens					Average	S.D. *	COV **
	1	2	3	4	5			
0.20%	0.24	0.26	0.22	0.25	0.22	0.24	0.02	7.02
0.40%	0.38	0.29	0.32	0.34	0.27	0.32	0.04	13.06
0.60%	0.62	0.54	0.41	0.83	0.65	0.61	0.16	25.58
0.80%	0.47	0.62	0.45	0.56	0.40	0.50	0.09	17.76
1.00%	0.45	0.40	0.50	0.56	0.50	0.48	0.06	12.52

* Standard deviation, ** Coefficient of variation.

**Table 7:** Residual strength at *L*/600 (MPa).

Fiber Content	Specimens					Average	S.D. *	COV **
	1	2	3	4	5			
0.20%	0.30	0.34	0.38	0.26	0.31	0.32	0.05	14.83
0.40%	0.38	0.43	0.54	0.54	0.37	0.45	0.08	18.76
0.60%	0.86	0.73	0.73	1.01	0.81	0.83	0.12	14.01
0.80%	0.62	0.83	0.62	0.78	0.60	0.69	0.11	15.38
1.00%	0.62	0.60	0.61	0.73	0.54	0.62	0.07	11.11

* Standard deviation, ** Coefficient of variation.

**Table 8:** Flexural properties (average of five specimens for each mix design).

Mix	First-Peak Strength (MPa)			Peak Strength (MPa)	Deflection					
					*L*/600			*L*/150		
					$P_{600}^{D}\;(\mathrm{kN})$	$f_{600}^{D}\;(\mathrm{MPa})$	$P_{150}^{D}\;(\mathrm{kN})$	$f_{150}^{D}\;(\mathrm{MPa})$	Tb (Nmm)	*f_e_* (MPa)
PP-0.0	0.47	0.03	6.80	0.47	-	-	-	-	-	-
PP-0.2	0.54	0.10	18.19	0.54	1.08	0.32	0.82	0.24	2089	0.30
PP-0.4	0.58	0.10	17.03	0.58	1.54	0.45	1.09	0.32	2901	0.42
PP-0.6	0.78	0.07	8.54	0.84	2.86	0.83	2.11	0.61	5210	0.75
PP-0.8	0.58	0.10	16.80	0.74	2.37	0.69	1.67	0.50	4312	0.62
PP-1.0	0.57	0.07	11.52	0.63	2.23	0.62	1.65	0.48	3992	0.57

**Table 9:** Model summary.

Model	Unstandardized Coefficients		Standardized Coefficients	t	Sig. (*p*-Value)
	B	Std. Error	Beta		
(Constant)	−0.268	0.085		−3.169	0.004
First Peak Strength (*f*_1_)	0.593	0.169	0.275	3.503	0.002
Fiber Content (*W_f_)*	1.066	0.139	1.422	7.692	0.000
Fiber Content Cubed (*W_f_^3^)*	−0.573	0.125	−0.803	−4.598	0.000
R	0.949				
R Square	0.900				
Adjusted R Square	0.889				
Std. Error of the Estimate	0.08695				

## References

[1] Jaquin P., Augarde A. (2012). Earth Building: History, Science and Conservation.

[2] Obonyo E., Tate D., Sika V., Tia M. (2010). Advancing the Structural Use of Earth-based Bricks: Addressing Key Challenges in the East African Context. Sustainability.

[3] Danso H., Martinson B., Ali M., Mant C. (2015). Performance characteristics of enhanced soil blocks: A quantitative review. Build. Res. Inf..

[4] Donkor P., Obonyo E. (2015). Earthen construction materials: Assessing the feasibility of improving strength and deformability of compressed earth blocks using polypropylene fibers. Mater. Des..

[5] Egenti C., Khatib J., Oloke D. (2014). Conceptualisation and pilot study of shelled compressed earth block for sustainable housing in Nigeria. Int. J. Sustain. Built Environ..

[6] Rigassi V. (1995). Compressed Earth Blocks Volume 1. Manual of Production.

[7] Morel J.-C., Pkla A., Walker P. (2007). Compressive strength testing of compressed earth blocks. Constr. Build. Mater..

[8] Hazeltine B., Bull C. (1999). Appropriate Technology: Tools, Choices and Implications.

[9] Akubue A. (2000). Appropriate Technology for Socioeconomic Development in Third World Countries. J. Technol. Stud..

[10] Adam E.A., Agib A. (2021). Compressed Stabilised Earth Block Manufacture in Sudan.

[11] United Nations Educational, Scientific and Cultural Organization (UNESCO) Engineering: Issues, Challenges and Opportunities for Development. Report. http://unesdoc.unesco.org/images/0018/001897/189753e.pdf.

[12] Morton T. (2008). Earth Masonry: Design and Construction Guidelines.

[13] Minke G. (2009). Building with Earth: Design and Technology of a Sustainable Architecture.

[14] Obonyo E., Donkor P., Matta F., Erdogmus E. Challenges, Opportunities, and Solutions in Low-cost Building Envelopes: A Case Study of Low-strength Masonry Systems. Proceedings of the Construction Research Congress.

[15] Donkor P., Obonyo E., Matta F., Erdogmus E. Effect of Polypropylene Fiber Length on the Flexural and Compressive Strength of Compressed Stabilized Earth Blocks. Proceedings of the Construction Research Congress.

[16] Donkor P. (2014). Sustainable and Resilient Earthen Masonry Systems: Enhancing Strength Properties and Flexural Performance using Polypropylene Fibers. Ph.D. Thesis.

[17] Donkor P., Obonyo E. (2016). Compressed soil blocks: Influence of fibers on flexural properties and failure mechanism. Constr. Build. Mater..

[18] Jayasinghe C., Mallawaarachchi R. (2009). Flexural strength of compressed stabilized earth masonry materials. Mater. Des..

[19] ASTM (2010). Standard Guide for Design of Earthen Wall Building Systems.

[20] New Mexico Construction Bureau (NMCB) (2009). New Mexico Adobe and Rammed Earth Building Code.

[21] Namango S. (2006). Development of Cost-Effective Earthen Building Material for Housing Wall Construction: Investigations into the Properties of Compressed Earth Blocks Stabilized with Sisal Vegetable Fibers, Cassava Powder and Cement Compositions. Ph.D. Thesis.

[22] Deboucha S., Hashim R.R. (2011). A review on bricks and stabilized compressed earth blocks. Sci. Res. Essays.

[23] Obonyo E. (2011). Optimizing the Physical, Mechanical and Hygrothermal Performance of Compressed Earth Bricks. Sustainability.

[24] Villamizar M.C.N., Araque V.S., Reyes C.A.R., Silva R.S. (2012). Effect of the addition of coal-ash and cassava peels on the engineering properties of compressed earth blocks. Constr. Build. Mater..

[25] Yu H., Zheng L., Yang J., Yang L. (2015). Stabilised compressed earth bricks made with coastal solonchak. Constr. Build. Mater..

[26] Alam I., Naseer A., Shah A. (2015). Economical stabilization of clay for earth buildings construction in rainy and flood prone areas. Constr. Build. Mater..

[27] Reddy B.V., Lal R., Rao K.S.N. (2007). Optimum Soil Grading for the Soil-Cement Blocks. J. Mater. Civ. Eng..

[28] Reddy B.V., Latha M. (2014). Influence of soil grading on the characteristics of cement stabilised soil compacts. Mater. Struct..

[29] Elenga R.G., Mabiala B., Ahouet L., Goma-Maniongui J., Diris G.F. (2011). Characterization of Clayey Soils from Congo and Physical Properties of their Compressed Earth Blocks Reinforced with Post-Consumer Plastic Wastes. Geomaterials.

[30] Taallah B., Guettala A., Guettala S., Kriker A. (2014). Mechanical properties and hygroscopicity behavior of compressed earth block filled by date palm fibers. Constr. Build. Mater..

[31] Mcgregor F., Heath A., Fodde E., Shea A. (2014). Conditions affecting the moisture buffering measurement performed on compressed earth blocks. Build. Environ..

[32] Reddy B.V.V., Gupta A. (2006). Tensile bond strength of soil-cement block masonry couplets using cement-soil mortars. J. Mater. Civ. Eng..

[33] Kitingu S.H. (2009). Design of Interlocking Bricks for Enhanced Wall Construction Flexibility, Alignment Accuracy and Load Bearing. Ph.D. Thesis.

[34] Bland D.W. (2011). In-Plane Cyclic Shear Performance of Interlocking Compressed Earth Block Walls. Master’s Thesis.

[35] Stirling B.J. (2011). Flexural Behavior of Interlocking Compressed Earth Block Shear Walls Subjected to In-Plane Loading. Master’s Thesis.

[36] Herskedal N.A. (2012). Investigation of Out-of-Plane Properties of Interlocking Compressed Earth Block Walls. Master’s Thesis.

[37] Tennant A.G., Foster C.D., Reddy B.V.V. (2013). Verification of Masonry Building Code to Flexural Behavior of Cement-Stabilized Soil Block. J. Mater. Civ. Eng..

[38] Qu B., Stirling B., Jansena D., Bland D., Laursen P. (2015). Testing of flexure-dominated interlocking compressed earth block walls, Constr. Build. Mater..

[39] Kibert C.J. (2003). Deconstruction: The start of a sustainable materials strategy for the built environment. UNEP Indus. Environ..

[40] Mortenson Center in Engineering for Developing Communities (MCEDC) Mortenson Center Introduces Sustainable Housing Solution on Crow Reservation. http://www.colorado.edu/engineering/academic-programs/mortenson-center-introduces-sustainable-housing-solution-crow-reservation.

[41] Crow Agency The Apsaalooke Nation Housing Authority’s Good Earth Lodges Project. http://sustainablenativecommunities.org/wp-content/uploads/2013/07/130611_13_CS-HUD-Good-Earth-Lodges.pdf.

[42] United Nations Centre for Human Settlements (1992). Earth Construction Technology.

[43] Subramaniaprasad C.K., Abraham B.M., Nambiar E.K.K. (2014). Influence of Embedded Waste-Plastic Fibers on the Improvement of the Tensile Strength of Stabilized Mud Masonry Blocks. J. Mater. Civ. Eng..

[44] American Concrete Institute (ACI) (1996). State-of-The-Art Report on Fiber Reinforced Concrete.

[45] Segetin M., Jayaraman K., Xu X. (2007). Harakeke reinforcement of soil–cementbuilding materials: Manufacturability and properties. Build. Environ..

[46] Manjunath K.R., Venugopal G., Rudresh A.N. (2013). Effect of random inclusion of sisal fibre on strength behavior of black cotton soil. Int. J. Adv. Res. Technol..

[47] Khedari J., Watsanasathaporn P., Hirunlabh J. (2005). Development of Fibre-Based Soil–Cement Block with Low Thermal Conductivity. Cem. Concr. Compos..

[48] Danso H., Martinson B., Ali M., Williams J. (2015). Physical, mechanical and durability properties of soil building blocks reinforced with natural fibres. Constr. Build. Mater..

[49] Bouhicha M., Aouissi F., Kenai S. (2005). Performance of composite soil reinforced with barley straw. Cem. Concr. Compos..

[50] Islam M., Iwashita K. (2010). Earthquake resistance of adobe reinforced by low cost traditional materials. J. Nat. Dis. Sci..

[51] Harshita Bairagi R.K.Y., Jain R. (2014). Effect of jute fibres on engineering properties of lime treated black cotton soil. Int. J. Adv. Res. Technol..

[52] Al-Salem S.M., Lettirei P., Baeyens J. (2009). Recycling and Recovery Routes of Plastic Solid Waste (PSW): A Review. Waste Manag..

[53] Heathcote K.A. (2002). An Investigation into the Erodability of Earth Wall Units. Ph.D. Thesis.

[54] Exelbirt J. (2011). Characterizing Compressed Earth Bricks Based on Hygrothermal Aging and Wind-Driven Rain Erosion. Master’s Thesis.

[55] Atahan H.N., Pekmezci B.Y., Tuncel E.Y. (2013). Behavior of PVA Fiber-Reinforced Cementitious Composites under Static and Impact Flexural Effects. J. Mater. Civ. Eng..

[56] Binici H., Aksogan O., Shah T. (2005). Investigation of fiber reinforced mud brick as building material. Constr. Build. Mater..

[57] Wilson A., Abolmaali A. (2013). Comparison of Material Behavior of Steel and Synthetic Fibers in Dry-Cast Application. Transp. Res. Rec. J. Transp. Res. Board.

[58] Maher M.H., Ho Y.C. (1994). Mechanical Properties of Kaolinite/Fiber Soil Composite. J. Geotech. Eng..

[59] Consoli N.C., Prietto P.D.M., Ulbrich L.A. (1998). Influence of Fiber and Cement Addition on Behavior of Sandy Soil. J. Geotech. Geoenviron. Eng..

[60] Consoli N.C., Vendruscolo M.A., Fonini A., Rosa F.D. (2009). Fiber reinforcement effects on sand considering a wide cementation range. Geotext. Geomembr..

[61] Tang C.-S., Shi B., Gao W., Chen F., Cai Y. (2007). Strength and mechanical behavior of short polypropylene fiber reinforced and cement stabilized clayey soil. Geotext. Geomembr..

[62] Jadhao P.D., Nagarnaik P.B. Performance Evaluation of Fiber Reinforced Soil—Fly Ash Mixtures. Proceedings of the 12th International Conference of International Association for Computer Methods and Advances in Geomechanics (IACMAG).

[63] Danso H., Martinson B., Ali M., Williams J. (2015). Effect of fibre aspect ratio on mechanical properties of soil building blocks. Constr. Build. Mater..

[64] Wilson A., Abolmaali A. (2014). Performance of Synthetic Fiber-Reinforced Concrete Pipes. J. Pipeline Syst. Eng. Pr..

[65] ASTM (2010). Standard Test. Method for Flexural Performance of Fiber-Reinforced Concrete (Using Beam with Third-Point Loading).

[66] Jamsawang P., Voottipruex P., Horpibulsuk S. (2015). Flexural Strength Characteristics of Compacted Cement-Polypropylene Fiber Sand. J. Mater. Civ. Eng..

[67] Mehta P.K., Monteiro P.J.M. (2006). Concrete, Microstructure, Properties and Materials.

[68] Sukontasukkul P., Jamsawang P. (2012). Use of steel and polypropylene fibers to improve flexural performance of deep soil–cement column. Constr. Build. Mater..

[69] Lin C., Kayali O., Morozov E., Sharp D.J. (2014). Influence of fibre type on flexural behaviour of self-compacting fibre reinforced cementitious composites. Cem. Concr. Compos..

[70] Prasad C.K.S., Nambiar E.K.K., Abraham B.M. (2012). Plastic Fiber Reinforced Soil Blocks as a Sustainable Building Material. Int. J. Adv. Res. Technol..

[71] Eko R.M., Offa E.D., Ngatcha T.Y., Minsili L.S. (2012). Potential of salvaged steel fibers for reinforcement of unfired earth blocks. Constr. Build. Mater..

[72] Walker P. (1995). Strength, durability and shrinkage characteristics of cement stabilised soil blocks. Cem. Conc. Compos..

[73] Walker P. (2004). Strength and erosion characteristics of earth blocks and earth block masonry. J. Mater. Civ. Eng..

[74] Arisoy B., Wu H. (2008). Material characteristics of high performance lightweight concrete reinforced with PVA. Constr. Build. Mater..

